# Dissociative and Epileptic seizures: how to distinguish them?

**DOI:** 10.1192/j.eurpsy.2022.1222

**Published:** 2022-09-01

**Authors:** A. Certo, J. Rodrigues, O. Nombora, M. Basto, E. Mendes

**Affiliations:** Vila Nova de Gaia Hospital Center, Psychiatry And Mental Health Service, Vila Nova de Gaia, Portugal

**Keywords:** epileptic seizures, dissociative disorders, differential diagnosis, Dissociative seizures

## Abstract

**Introduction:**

Dissociative seizures (DS) are classified as dissociative convulsions within the group of dissociative disorders. Although they share many features with epileptic seizures (ES), they are not a consequence of abnormal brain discharges and may be related to psychogenic causes. DS represent a common diagnostic and are often confounded with ES.

**Objectives:**

The aim of this study is to review the current evidence about the differential diagnosis between DS and ES.

**Methods:**

We conducted a non-sytematic review on the topic, using Pubmed/Medline database.

**Results:**

Studies emphasize a correct diagnosis before treatment of seizures. DS and ES respond differently to anticonvulsant medication and early or incorrect prescription of can even exacerbate DS. Clinical features and a neuropsychiatric history can also help. The presence of a dissociative “stigmata”, such as unexplained sensory loss, may support a non-epileptic diagnosis. EEG videorecording method is the gold standard diagnosis for DS, however often displays rhythmic movement artifacts that may resemble seizure activity and confound the interpretation. The absence of ictal EEG discharges characteristic of epilepsy is a sign of DS. However, this may not be true for some partial ES, particularly those from temporal lobes, whom also tend to report shorter duration of seizures, whereas patients with DPD often describe experiences lasting for hours or longer.

**Conclusions:**

Distinguish DS from ES can be challenging. However, there are features that can help in the differential diagnosis. A correct diagnosis is essential for an adequate therapeutic approach, better prognosis, reduction of medical costs and also a referral to the right medical specialty.

**Disclosure:**

No significant relationships.

